# Long-term cognitive impairment after acute respiratory distress syndrome: a review of clinical impact and pathophysiological mechanisms

**DOI:** 10.1186/s13054-019-2626-z

**Published:** 2019-11-12

**Authors:** Cina Sasannejad, E. Wesley Ely, Shouri Lahiri

**Affiliations:** 1 Division of Neurocritical Care and Emergency Neurology, Department of Neurology, Yale School of Medicine, New Haven, CT USA; 2 Critical Illness, Brain Dysfunction, Survivorship (CIBS) Center, Department of Pulmonary and Critical Care Medicine, Veteran’s Affairs Tennessee Valley Geriatric Research Education and Clinical Center (GRECC), Vanderbilt University School of Medicine, Nashville, TN USA; 3 Division of Neurocritical Care, Department of Neurology, Cedars-Sinai Medical Center, 127 S. San Vicente Blvd, AHSP Building, Suite A6600, A8103, Los Angeles, CA 90048 USA; 4 Division of Neurocritical Care, Department of Neurosurgery, Cedars-Sinai Medical Center, 127 S. San Vicente Blvd, AHSP Building, Suite A6600, A8103, Los Angeles, CA 90048 USA; 5 Division of Neurocritical Care, Department of Biomedical Sciences, Cedars-Sinai Medical Center, 127 S. San Vicente Blvd, AHSP Building, Suite A6600, A8103, Los Angeles, CA 90048 USA

**Keywords:** ARDS, Cognitive impairment, Outcomes, ICU delirium, Blood-brain barrier, Inflammation, Mechanical ventilation, Pathophysiological mechanisms

## Abstract

Acute respiratory distress syndrome (ARDS) survivors experience a high prevalence of cognitive impairment with concomitantly impaired functional status and quality of life, often persisting months after hospital discharge. In this review, we explore the pathophysiological mechanisms underlying cognitive impairment following ARDS, the interrelations between mechanisms and risk factors, and interventions that may mitigate the risk of cognitive impairment. Risk factors for cognitive decline following ARDS include pre-existing cognitive impairment, neurological injury, delirium, mechanical ventilation, prolonged exposure to sedating medications, sepsis, systemic inflammation, and environmental factors in the intensive care unit, which can co-occur synergistically in various combinations. Detection and characterization of pre-existing cognitive impairment imparts challenges in clinical management and longitudinal outcome study enrollment. Patients with brain injury who experience ARDS constitute a distinct population with a particular combination of risk factors and pathophysiological mechanisms: considerations raised by brain injury include neurogenic pulmonary edema, differences in sympathetic activation and cholinergic transmission, effects of positive end-expiratory pressure on cerebral microcirculation and intracranial pressure, and sensitivity to vasopressor use and volume status. The blood-brain barrier represents a physiological interface at which multiple mechanisms of cognitive impairment interact, as acute blood-brain barrier weakening from mechanical ventilation and systemic inflammation can compound existing chronic blood-brain barrier dysfunction from Alzheimer’s-type pathophysiology, rendering the brain vulnerable to both amyloid-beta accumulation and cytokine-mediated hippocampal damage. Although some contributory elements, such as the presenting brain injury or pre-existing cognitive impairment, may be irreversible, interventions such as minimizing mechanical ventilation tidal volume, minimizing duration of exposure to sedating medications, maintaining hemodynamic stability, optimizing fluid balance, and implementing bundles to enhance patient care help dramatically to reduce duration of delirium and may help prevent acquisition of long-term cognitive impairment.

## Introduction

Cognitive decline following acute respiratory distress syndrome (ARDS) complicates recovery from critical illness, particularly among elderly patients with pre-existing cognitive impairment. Although the exact pathophysiological mechanisms are unknown, it is widely believed that neurological injury due to acute systemic inflammatory dysregulation or impairments in cerebrovascular hemodynamics contribute to cognitive decline after ARDS. In this article, we review epidemiology, risk factors, putative pathophysiological mechanisms, and possible therapeutic approaches to minimize cognitive decline after ARDS.

## Background and epidemiology

### Cognitive impairment in survivors of acute respiratory distress syndrome: clinical burden and long-term sequelae

Acute respiratory distress syndrome (ARDS) affects 200,000 patients per year in the USA, accounting for 10.4% of ICU admissions and 25–40% mortality risk [1, 2]. ARDS survivors experience a high prevalence of cognitive impairment: 70–100% at hospital discharge, 46–80% at 1 year, and 20% at 5 years [3, 4]. ARDS survivors score significantly lower on standardized quality of life assessments compared to severity-matched controls at 6- and 12-month follow-up [5, 6]. ARDS survivors also experience higher overall healthcare costs, exercise limitations, and persistent psychological and physical disability despite lung function recovery at 5-year follow-up [7]. Upon 1-year follow-up, survivors demonstrate impaired executive function and short-term memory and increased rates of anxiety and depression [1, 8], in addition to post-traumatic stress disorder [9]. Neurocognitive testing of ARDS survivors at 2-year follow-up reveals residual emotional and cognitive sequelae in nearly half of patients [10]. In addition to depression and anxiety, testing shows impairments in executive function, learning, and memory, with 50% of those affected performing below the 6th percentile on multiple instruments [10]. Selected clinical studies relevant to the understanding of post-ARDS cognitive impairment are summarized in Table 1.

**Table 1:** Selected clinical studies investigating post-ARDS cognitive impairment

Author(s)	Year	Methodology	Results/conclusions
Davidson et al.	1999	Prospective cohort (*n* = 146)	Patients who survived ARDS experience significantly reduced quality of life following discharge compared to critically ill patients without ARDS
Hopkins et al.	1999	Prospective cohort (*n* = 62)	Survivors of ARDS demonstrate cognitive impairments in memory, attention, concentration, and processing speed: 100% at discharge and 78% at 1 year after discharge
Contant et al.	2001	Observational (*n* = 161)	ARDS following severe head injury results in severe intracranial hypertension. Targeting intracranial pressure rather than cerebral blood flow improves outcomes
Georgiadis et al.	2001	Prospective interventional (*n* = 20)	In patients with acute stroke receiving mechanical ventilation, changes in cerebral perfusion pressure are mediated by mean arterial pressure rather than by positive end-expiratory pressure. Positive end-expiratory pressure does not increase intracranial pressure as long as hemodynamic stability is maintained
Holland et al.	2003	Prospective cohort (*n* = 137)	In patients with traumatic brain injury, ARDS independently predicts mortality and is associated with worse long-term neurological outcome
Ely et al.	2004	Prospective cohort (*n* = 275)	Delirium independently predicts higher mortality and longer hospital stay among patients treated with mechanical ventilation
Mascia et al.	2005	Prospective interventional (*n* = 12)	Positive end-expiratory pressure does not affect intracranial pressure when inducing alveolar recruitment, but does lead to significant increases in PaCO_2_ and intracranial pressure when inducing alveolar hyperinflation
Muench et al.	2005	Prospective interventional (*n* = 10)	In hemodynamically unstable patients with severe subarachnoid hemorrhage, increases in positive end-expiratory pressure disturb cerebrovascular autoregulation, resulting in significant decreases in mean arterial pressure and regional cerebral blood flow
Mascia et al.	2007	Observational (*n* = 82)	High-tidal-volume mechanical ventilation is associated with the development of ARDS after severe brain injury
Fong et al.	2009	Secondary analysis of prospective cohort (*n* = 408)	Delirium accelerates cognitive decline in patients with probable or possible Alzheimer’s disease
Taccone et al.	2009	Observational (*n* = 21)	Septic shock impairs cerebral autoregulation in patients with septic shock, particularly with concurrent hypercapnia
Janz et al.	2010	Retrospective cohort (*n* = 7 from database of 379)	Brain autopsy of patients with ICU delirium shows hypoxic ischemic damage in the hippocampus, suggesting a link between ICU delirium and long-term cognitive impairment
van den Boogard et al.	2011	Exploratory observational (*n* = 100)	The underlying mechanism of delirium may differ in patients with systemic inflammation versus patients without systemic inflammation and is mediated by different cytokines for each mechanism
Mikkelsen et al.	2012	Prospective cohort (*n* = 102)	Survivors of ARDS 1 year following discharge demonstrate a confluence of cognitive impairment, psychiatric sequelae, and diminished quality of life. Hypoxemia and conservative fluid management are associated with these long-term impairments
Elmer et al.	2013	Retrospective cohort (*n* = 697)	High-tidal-volume mechanical ventilation in patients with intracerebral hemorrhage is associated with the development of ARDS and increased mortality
Pandharipande et al.	2013	Prospective cohort (*n* = 821)	At 12-month follow-up after discharge, 1/4 of patients who had been critically ill demonstrate cognitive impairment similar in severity to that seen in mild Alzheimer’s disease, and 1/3 similar in severity to that seen in traumatic brain injury
Needham et al.	2014	Prospective cohort (*n* = 203)	At 6- and 12-month follow-up, ARDS survivors demonstrated impairments in 6-min walk distance and physical function outcomes. Minimizing the duration of intensive care and corticosteroid use may reflect modifiable risk factors
Girard et al.	2018	Prospective cohort (*n* = 1040 enrolled, *n* = 586 follow-up)	Patients with ARDS, septic shock, or both experience multiple subtypes of delirium associated with long-term cognitive impairment at 3- and 12-month follow-up, including hypoxic, septic, unclassified, and sedative-associated delirium. The durations of these delirium subtypes predict worse cognitive function at 12-month follow-up, particularly sedative-associated delirium

Ascertaining the true cognitive impact of ARDS requires distinction between patients with pre-ARDS cognitive impairment and patients who develop new cognitive symptoms after ARDS. Although established instruments, such as the Mini-Mental State Examination (MMSE), identify general memory impairment at a single point in time, two limitations arise in ICU populations: incomplete patient participation and the inability to distinguish between delirium, dementia, or the simultaneous presence of both [11]. These limitations led to the development of instruments more sensitive in discerning between different types and timescales of cognitive impairment. The Confusion Assessment Method for Intensive Care Unit patients (CAM-ICU) shows high sensitivity and specificity in detecting delirium in mechanically ventilated patients, incorporating questions addressing the onset of mental status change, inattention, disorganized thinking, and changes in the patient’s level of consciousness [12]. Other instruments have been developed to evaluate for pre-existing cognitive deficits: the Modified Blessed Dementia Rating Scale (mBDRS) and the Informant Questionnaire on Cognitive Decline in the Elderly (IQCODE) [13]. These instruments more sensitively detect pre-existing cognitive impairment by incorporating questions about activities of daily living and practical examples of learning or memory in the patient’s pre-morbid daily life experiences, thereby reducing the burden of direct participation expected from the patient or allowing completion by proxy [11, 14]. The mBDRS assesses for dementia across 11 items encompassing memory, money management, navigation, activities of daily living, and personality changes, while the IQCODE measures longitudinal cognitive decline across 16 items encompassing memory, tool utilization, learning, decision-making, and problem solving [11, 13]. While these instruments show high overall agreement, the IQCODE demonstrates greater sensitivity, while the mBDRS is less dependent on access to a proxy [11, 13]. In the long-term follow-up of survivors of ARDS, the MMSE shows poor sensitivity in detecting cognitive impairment and weak-to-moderate correlation with neuropsychological testing [15]. Implementation and development of specialized cognitive assessment tools may help better guide decisions regarding capacity and medications, in addition to improving our understanding of the significant impact of cognitive decline after ARDS.

### ARDS in brain injury

ARDS in the context of acute brain injury constitutes a distinct clinical scenario from other types of ARDS, characterized by earlier sympathetic activation and potential interactions between positive pressure ventilation, cerebral autoregulatory, and microcirculatory function [16]. ARDS independently predicts mortality and poor neurological outcome in patients with acute brain injury, particularly in traumatic brain injury or intracerebral hemorrhage [17]. Stevens and Puybasset posited a “brain-lung-brain axis” by which severe neurological injury from traumatic brain injury, subarachnoid hemorrhage, or status epilepticus can provoke concurrent pulmonary injury, in turn worsening overall neurocognitive outcomes [18]. Up to 25% of patients with severe brain injury develop ARDS and up to 50% develop pulmonary edema, the former increasing the likelihood of death or vegetative state threefold [19, 20]. Patients with brain injury incur the greatest risk for developing ARDS in the initial 2–3 days after injury and approximately 1 week after injury [21, 22]. Sympathetic activation, systemic inflammation, pulmonary insufficiency, and shock contribute to the risk of ARDS early in hospitalization [21, 22]. The second peak of ARDS risk reflects ventilator-associated pneumonia and sepsis [21, 22]: comatose patients experience an increased frequency of pneumonia between hospital days 4–7 and are at risk for aspiration of oropharyngeal secretions during and after intubation [23]. An observational study of patients with severe traumatic brain injury identified cerebral midline shift exceeding 5 mm and prior drug abuse as risk factors for ARDS [19]. Furthermore, patients with concurrent ARDS and brain injury who experienced worse long-term cognitive outcome tended to demonstrate lower systemic blood pressure, higher intracranial pressure, and lower cerebral perfusion pressure [19]. Among patients with intracerebral hemorrhage, 27% develop ARDS, with high-tidal-volume mechanical ventilation constituting the greatest risk factor, followed by positive fluid balance and blood transfusion [24]. Despite this, existing prognostic models for acute brain injury largely do not include ARDS as a variable that may independently limit the extent of neurocognitive recovery.

## Risk factors for cognitive decline

**Fig. 1:**
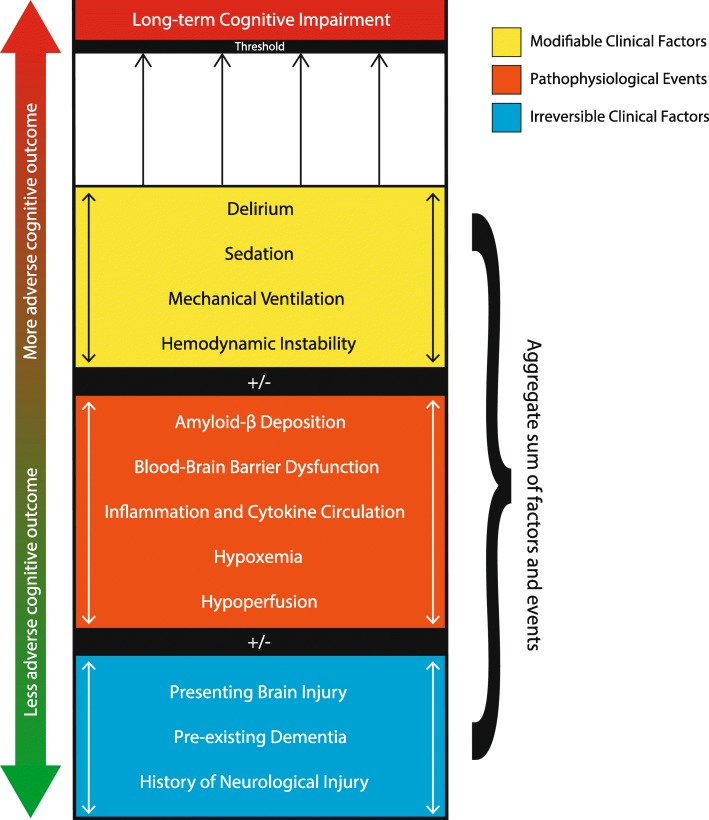
Confluence of clinical risk factors and pathophysiological events culminating in cognitive impairment following ARDS and brain injury. Combinations of irreversible clinical risk factors, pathophysiological events, and modifiable clinical risk factors, each occurring to varying extents, produce an aggregate sum of risk for long-term cognitive impairment. Cognitive outcomes reflect a continuum up to a threshold beyond which a patient is likely to experience an adverse outcome, defined as long-term cognitive impairment. The aggregate sum of these factors can bring the patient’s risk for long-term impairment closer toward this threshold (the upward trajectory indicated by the red arrow); however, minimization of modifiable clinical factors can bring the aggregate sum further away from the threshold, promoting a less adverse cognitive outcome (the downward trajectory indicated by the green arrow)

Risk factors for long-term cognitive impairment comprise a combination of irreversible clinical factors, potentially modifiable clinical complications of provider interventions, and pathophysiological events that may occur in the natural history of ARDS in brain injury. Figure 1 illustrates the confluence of these factors and how they may culminate in an adverse long-term cognitive outcome. The heterogeneity of ARDS etiology and severity can expose patients to varying balances of these factors: for instance, ARDS of a pulmonary etiology may expose patients to more severe hypoxemia, whereas ARDS related to sepsis may expose patients to more severe inflammatory activation, while overall disease severity can impact length of stay.

### Pre-existing cognitive impairment and the interface of delirium and dementia

Pre-existing cognitive impairment is a risk factor for cognitive decline after critical illness [25, 26], though data are limited by under-recognition of pre-existing cognitive impairment [14] or exclusion of patients with pre-existing cognitive impairment from longitudinal follow-up studies [1, 6]. Alzheimer’s disease, characterized by cerebral accumulation and deposition of the amyloid-β peptide, is the most common type of cognitive impairment [27, 28]. Although one third of elderly patients admitted to the intensive care unit have pre-existing cognitive impairment, this history is often unknown to their medical teams [14], in turn precluding comparisons of pre-morbid versus longitudinal cognitive performance. Among patients confirmed not to have pre-existing dementia, one study found hospitalization itself significantly associated with a greater likelihood of developing dementia, along a temporal pattern of abrupt, rather than gradual, cognitive decline [29].

Delirium during critical illness predicts long-term cognitive decline after ARDS [1, 4, 26], and longer durations of delirium in critically ill patients predict more severe cognitive impairment at 1-year follow-up [30]. The closely intertwined relationship between delirium and pre-existing cognitive impairment raises a physiological question: does long-term cognitive impairment following ARDS reflect a reduction in the threshold for developing delirium due to underlying Alzheimer’s pathology, or does delirium contribute independently to this end? It is known that pre-existing cognitive impairment is a key underlying risk factor for delirium, which affects as many as 70–87% of critically ill patients [31, 32]. Particularly among the elderly, ICU delirium can persist during hospitalization following transfer from the ICU in 40–50% of patients, commonly with incomplete resolution by discharge [33]. A meta-analysis of long-term sequelae of delirium in elderly patients found increased risk of developing dementia within 3–5 years of discharge, with an odds ratio of 12.52 (95% CI 1.86–84.21) [34]. Patients with Alzheimer’s disease, when hospitalized, are three times as likely as adults without dementia to experience delirium; cognitive deficits can persist up to 5 years after discharge [25]. Clinically, patients with Alzheimer’s disease who experience delirium suffer accelerated cognitive decline beyond the natural course of dementia alone, with twofold increases in the slope of decline [35]. Recent pathology-based studies corroborate delirium-associated acceleration of cognitive decline independent of pre-existing dementia pathology [36].

### Delirium subtypes and pathways

Studies of the pathophysiology of short- and long-term cognitive impairment reflect similarities and differences between delirium and dementia pathways, in which pre-existing pathophysiology diminishes the reserve with which to face acute insults. Shared mechanistic features of dementia and delirium include synaptic disconnection resulting from the loss of presynaptic terminals, diminished cholinergic activity leading to impaired arousal and inattention, and microglial activation perpetuating systemic inflammation [37]. Girard et al. classify the phenotypes of delirium in the intensive care unit into five subtypes: sedative-associated, hypoxic, septic/inflammatory, metabolic, and unclassified [30]. Among these, the duration of metabolic delirium is not associated with long-term adverse cognitive outcomes [30]. Pathophysiologically, MacLullich et al. classify the etiologies of delirium into two categories: direct brain insults—primary insults such as hemorrhage, hypoxia, hypoperfusion, or drugs—versus aberrant stress responses—the dysfunction of ordinarily adaptive responses to acute systemic stressors such as infection or trauma [38]. Differences in serum markers between patients with inflammatory versus non-inflammatory delirium suggest activation of multiple possible pathways: a systemic inflammation pathway associated with IL-8 elevation among patients with inflammation, and an alternative pathway characterized by elevation of amyloid-β and IL-10 in patients without inflammation, suggesting activation of existing pathways of underlying cognitive impairment [39]. Individual pathophysiological mechanisms may be associated with multiple delirium subtypes in which a single phenotype dominates, and these relationships remain an active area of investigation.

### ARDS and hypoxemia: short-term and long-term effects

Profound hypoxemia is one of the cardinal features of ARDS. While, in the short-term, this can predispose patients to hypoxic delirium phenotypes [30], lower PaO_2_ levels are associated with long-term cognitive impairment at 12-month follow-up, particularly in the domains of executive function and psychomotor tasks [1, 40].

### Inflammation and septic delirium

The production of cytokines TNF-α, IL-6, IL-1α, and IL-1β can produce a constellation of behaviors termed “sickness behavior,” comprising impaired concentration, malaise, diminished motivation, psychomotor retardation, and depression [41], corresponding with septic delirium. In one study, this state was found to be associated with recruitment of additional brain structures in order to maintain the same level of cognitive performance during systemic inflammation [42]. Existing brain injury enhances vulnerability to inflammation: in mouse models of neurodegenerative disease, lipopolysaccharide in brain-injured versus control mice induces compounded cognitive deficits on maze-learning tasks greater than exaggerated sickness behavior alone [43].

### Mechanical ventilation and inflammatory and sedative-associated delirium

Mechanical ventilation is independently associated with persistent cognitive impairment, diminished quality of life, and depression [44]. One third of mechanically ventilated patients perform abnormally on neurocognitive testing at 6 months, comprising deficits in visuo-construction, visual memory, psychomotor speed, and verbal fluency [44]. Accelerated Alzheimer’s disease-type pathophysiology can follow short-term high-tidal-volume mechanical ventilation in mice, including impaired β-amyloid clearance, increased inflammation mediated by TNF-α and IL-6, and altered blood-brain barrier permeability [45]. Prolonged courses of mechanical ventilation in ARDS also expose patients to sedating medications and anesthesia. Among delirium subtypes, sedative-associated delirium is the most common and, with prolonged duration, associated with the greatest degree of long-term cognitive impairment at 12 months following discharge [30]. Among sedative types, benzodiazepines impart the greatest risk for delirium, while dexmedetomidine has been associated with lower risk for delirium [46]; however, data and practice choices are limited, as most intensive care unit patients are treated with multiple sedatives [30]. The effect of paralytic exposure on long-term cognitive outcomes is unknown [3]. Continuous exposure to sedative medications over several days results in impaired sedative clearance, in turn exacerbating delirium [30]. In addition to inducing acute-on-chronic cholinergic transmission dysfunction in elderly patients, transient axonal damage may represent another mechanism by which sedatives can contribute to long-term cognitive decline [47].

## Putative biological mechanisms

### Inflammation and hypoxemia

Animal studies of ARDS reflect a wide range of approaches, including murine and porcine models, chemical lung injury, ventilator-induced lung injury following mechanical ventilation, and combinations of lung injury with systemic inflammatory states, such as sepsis [48]. Selected animal studies relevant to the understanding of the mechanisms of post-ARDS cognitive impairment are summarized in Table 2. While ARDS models typically invoke a high-tidal-volume strategy, it is important to note that mechanical ventilation itself, even at low tidal volumes, can still cause lung injury [49].

**Table 2:** Selected animal studies investigating post-ARDS cognitive impairment

Author(s)	Year	Animal model	Results/conclusions
De la Torre et al.	1992	Rat	Chronic cerebrovascular insufficiency following ligation of the common carotid and left subclavian arteries in aged rats induces behavioral and cognitive impairments consistent with dementia
Pappas et al.	1996	Rat	Rats exposed to chronic reduction of cerebral blood flow following carotid artery ligation develop memory dysfunction and cell loss in the CA1 region of the hippocampus
Feldman et al.	1997	Rabbit	Positive end-expiratory pressure reduces intracranial compliance in rabbits
Wilson et al.	2003	Mouse	High-tidal-volume mechanical ventilation upregulates cytokines in mouse lungs
Altmeier et al.	2005	Mouse	Systemic inflammation simulated by lipopolysaccharide in mechanically ventilated mice induces cytokine-mediated lung injury in mechanically ventilated wild-type mice
Fries et al.	2005	Pig	Mechanically ventilated pigs exposed to hypoxemia with lung injury develop histopathologic changes in the CA1 region of the hippocampus not when exposed to the same degree of hypoxemia alone, suggesting lung injury as a mechanism of damage independent from hypoxemia
Semmler et al.	2005	Rat	Systemic inflammation induces apoptosis in the rat brain, particularly in the hippocampus
Wilson et al.	2005	Mouse	Pulmonary inflammation following high-tidal-volume mechanical ventilation in mice without underlying lung injury is mediated by TNF-α
Bickenbach et al.	2009	Pig	Low- versus high-tidal-volume mechanical ventilation improves cerebral tissue oxygenation in pigs
Wolthuis et al.	2009	Mouse	Mechanical ventilation even at lower tidal volumes causes lung injury in wild-type mice without history of lung disease
Bickenbach et al.	2011	Pig	Mechanically ventilated pigs exposed to hypoxemia with lung injury demonstrate trends toward elevated cytokines IL-6 and TNF-α in the CA1 region of the hippocampus versus mechanically ventilated pigs exposed to hypoxemia alone
Heuer et al.	2011	Pig	In mechanically ventilated pigs, ARDS results in elevations in TNF-α, IL-6, and IL-1β, which further increase in pigs with acute intracranial hypertension. The combination of ARDS and acute intracranial hypertension results in hippocampal damage. Acute intracranial hypertension induces lung injury and extravascular lung water
Imamura et al.	2011	Mouse	In a mouse model of septic encephalopathy, an IL-1β cytokine-mediated process disrupts the synaptic processing of long-term potentiation in the hippocampus
Davis et al.	2015	Mouse	Baseline neurodegeneration in mice increases the risk, duration, and severity of delirium
Shohami et al.	2016	Rat	TNF-α and IL-6 are detected in the contused hemisphere of rats soon after closed head injury, but not in healthy rats. TNF-α is detected as early as 1 h after injury and peaks at 4 h, whereas IL-6 is detected at 3–5 h and peaks at 8 h after injury
Lahiri et al.	2019	Mouse	High-tidal-volume mechanical ventilation simulates Alzheimer’s disease pathophysiology in transgenic Alzheimer’s disease and wild-type mice

Various mechanisms of ARDS-mediated neurological damage have been theorized, including hypoxemia and cytokine-mediated damage [50]. A porcine model of ARDS identified cytokine-mediated brain damage from lung injury, rather than hypoxemia, as the major pathophysiological contributor to hippocampal damage, specifically in CA1 and CA2 [50]. A subsequent study on pigs randomized to mechanical ventilation-induced ARDS versus hypoxia-only groups found greater cognitive impairment and trends toward increased hippocampal inflammation and systemic IL-6 and TNF-α expression among ARDS subjects, further corroborating the distinct pathophysiological roles of mechanical ventilation and concomitant cytokine release [51]. Among mechanical ventilation groups, low-tidal-volume ventilation is associated with improved brain tissue oxygenation and reduced cytokine release compared to high-tidal-volume groups [52].

Animal models also demonstrate that ARDS associated with acute brain injury differs in physiology, time course, and treatment from traditional ARDS: it occurs later and features sympathetic nervous system activation, which, in turn, can trigger neurogenic pulmonary edema secondary to increased alpha-adrenergic activity [53, 54].

ARDS is frequently triggered by sepsis, a proinflammatory condition characterized by elevated peripheral cytokines and cerebral hypoperfusion, culminating in multifactorial encephalopathy and end-organ damage [55]. Peripheral cytokine elevation alters blood-brain barrier metabolism by activating endothelial cells, while simultaneously impairing systemic and cerebral blood flow, altering glucose metabolism in the brain, and exposing patients to deleterious environmental factors in the course of treatment [55]. Cerebral autoregulation disruption in early septic shock further compounds systemic hypotension, as endotoxin-triggered inducible nitric oxide synthase production excessively vasodilates blood vessels, precluding appropriate modulation of vascular resistance [56]. Patients with concurrent sepsis and ARDS incur further risk for impaired cerebral autoregulation, as sepsis renders the cerebral autoregulatory mechanism more sensitive to P_a_CO_2_: in one study, 50% of septic patients with low P_a_CO_2_ lost autoregulation, rising to 100% with normal or high P_a_CO_2_ [56].

### Blood-brain barrier damage and amyloid-β clearance

The blood-brain barrier is a key neurobiological structure underlying cognitive function. Under normal physiological conditions, the blood-brain barrier transports amyloid-β protein from within neurons to the extracellular space, where it can be cleared by the glymphatic system [57–59]. Accumulated amyloid-β worsens blood-brain barrier dysfunction by increasing permeability, impairing transporter function, and modulating endothelial cell expression patterns, in turn perpetuating impairment in the clearance of amyloid-β and inflammatory cytokines [60]. Amyloid-β toxicity induces apoptosis in blood-brain barrier endothelial cells and downregulates tight junction proteins Zo-1, occludin, and claudin [61]. Hippocampal blood-brain barrier breakdown has been identified early in the disease course in transgenic Alzheimer’s disease mouse models, with breakdown preceding the detection of amyloid-β deposits, cerebral amyloid angiopathy, or behavioral changes [58]. Animal studies demonstrate a relationship between amyloid-β deposits and hippocampal damage, as intrahippocampal injection of amyloid-β in rats specifically induced apoptosis in the CA1 region of the hippocampus [62]. Amyloid-β impairs the memory-consolidation process of long-term potentiation in the rat hippocampus in a time- and concentration-dependent fashion [63], mechanistically comprising impairments in intracellular calcium homeostasis and NMDA receptor function [27].

Pathophysiological similarities between Alzheimer’s disease and the acute sequelae of high-tidal-volume mechanical ventilation in mice suggest a link between the mechanisms underlying cognitive damage in ARDS, Alzheimer’s disease, and delirium through a combination of inflammation and amyloid-β accumulation [45]. Mechanical ventilation increases cerebral TNF-α independently of serum TNF-α, suggesting that cerebral amyloid-β deposition in this setting may reflect a direct response to mechanical ventilation or pulmonary injury rather than sequelae of systemic inflammation [45].

Neuroimaging comparisons of ARDS survivors within a year of hospital discharge, versus healthy matched control patients, demonstrate accelerated cerebral and hippocampal atrophy [64]. This is consistent with autopsy findings from deceased intensive care unit patients who had experienced delirium, in which hippocampal hypoxic ischemic lesions were not only the most commonly identified abnormality, but were also found only in patients who had experienced ARDS [65]. Magnetic resonance imaging studies have identified hippocampal blood-brain barrier dysfunction preceding structural or functional phenotypes [66]. Post-mortem studies reflect consistency with these results, with evidence of microvascular damage and leukocyte infiltration at the sites of blood-brain barrier damage [66].

## Acute and chronic blood-brain barrier insults: linking risk factors and mechanisms

### Acute weakening, diminished reserve, cytokine circulation, and apoptosis

ARDS and concomitant systemic inflammation reflect a multifactorial array of simultaneous insults, comprising cytokine release, metabolic dysregulation, impaired cerebral perfusion, medication side-effects, and environmental stimuli including physical restraints and noise [55]. Peripheral cytokine release generates positive feedback, inciting further cytokine production through vagal tone increase, blood-brain barrier endothelial activation, and humoral system activation [55]. These processes collectively culminate in microglial activation, releasing additional cytokines, nitric oxide, and reactive oxidative species within the central nervous system [55]. Microglial activation occurs not only in systemic inflammation, but also in aging, as older rodents injected with peripheral *E. coli* lipopolysaccharide produced cytokines, primarily IL-1β, selectively in the hippocampus [67, 68]. A study investigating anatomic patterns of apoptosis in a mouse model of systemic inflammation identified the hippocampus as the most vulnerable region to Bax-mediated apoptotic cascade activation following nitric oxide synthase production downstream of microglial activation [69].

Anatomical studies of humans and rodents confirm the selective vulnerability of the CA1 hippocampal layer to ischemic injury in a mechanism thought to reflect glutamate excitotoxicity [70]. Mouse models of septic encephalopathy confirm the role of IL-1β in damaging learning and memory centers of the hippocampus, as hippocampal neurons expressing IL-1β demonstrated electrophysiological evidence of inhibition of long-term potentiation [71], thereby acting in a similar mechanism as amyloid-β-mediated impairment of learning and memory [72]. Existing blood-brain barrier damage secondary to amyloid-β accumulation is associated with diminished cerebral blood flow and impaired blood flow regulation in the elderly, secondary to impaired neurovascular coupling [73]. This, in turn, can render patients with Alzheimer’s disease susceptible to worsening cognitive dysfunction in the setting of systemic hypoperfusion and hypoxemia, which are common in ARDS [73].

The mechanistic relationships of brain and lung injuries reflect synergy rather than direct causality, as brain injury itself triggers cytokine production, including TNF-α and IL-6 [74]. Indeed, lungs harvested from rabbits that had undergone brain herniation were less resilient in tolerating high-pressure mechanical ventilation compared to sham craniostomy animals, suggesting potentiation of ARDS by systemic inflammation, in turn due to brain injury [75]. Baseline blood-brain barrier weakening, from chronic amyloid-β accumulation, renders patients with mild cognitive impairment and Alzheimer’s disease susceptible to increased hippocampal exposure to cytokines. Systemic inflammation, from ARDS and sepsis, in turn, perpetuates cytokine-mediated hippocampal damage by imparting acute-on-chronic blood-brain-barrier damage while simultaneously increasing systemic cytokine circulation.

Organ crosstalk in the setting of injurious mechanical ventilation is not limited to brain-lung interactions. Animal studies demonstrate lung-kidney and lung-gut interactions yielding insight into the pathogenesis of multi-organ dysfunction syndrome, as rabbits exposed to high-tidal-volume mechanical ventilation developed epithelial cell apoptosis in the kidney and small intestine [76]. In addition to cytokine-mediated damage and apoptosis, additional mechanisms of acute kidney injury associated with high-tidal-volume mechanical ventilation include renal blood flow redistribution, hypoperfusion from systemic hemodynamic changes, and metabolic disruption from blood gas changes [77]. Human studies corroborate this relationship, with a threefold increase in the risk of acute kidney injury among critically ill patients exposed to invasive mechanical ventilation (OR 3.58, 95% CI 1.85–6.92) [78].

### Positive pressure ventilation and amyloid-β accumulation

Despite benefits of positive end-expiratory pressure (PEEP) in improving oxygenation and alveolar recruitment, its application also introduces physiological risk: increased intrathoracic pressure from PEEP can impair both cerebral venous outflow and systemic venous return, resulting in simultaneously increased intracranial pressure and reduced cerebral perfusion pressure, respectively [16]. Animal studies have found increased intracranial pressure and decreased mean arterial pressure with increases in PEEP, in addition to reduced intracranial compliance [79]. In another study on a mouse model of stroke, increased PEEP reduced cerebral perfusion pressure by reducing mean arterial pressure, but had minimal effect on intracranial pressure when mean arterial pressure remained stable, reinforcing the importance of hemodynamic stability [79]. Human studies confirm the effects of PEEP in hemodynamically unstable patients, as PEEP up to 20 cm H_2_O was shown in one study to significantly decrease mean arterial pressure and cerebral blood flow [80]. Consequences of PEEP on cerebral microcirculation therefore may compound effects from other potential mechanisms of cognitive impairment in critically ill patients with both ARDS and brain injury, exacerbating both the cerebral hypoperfusion of sepsis and the impairment in amyloid-β clearance following impaired cerebral outflow in vulnerable patients. In another study in acutely brain-injured patients, PEEP leading to alveolar hyperinflation increased dead space and P_a_CO_2_, in turn resulting in arterial vasodilation and concomitantly increased intracranial pressure, with further worsening in the setting of diminished intracranial compliance [81]. The effects of PEEP on cerebrovascular hemodynamics, neurological injury, and cognitive outcomes remain largely unknown and represent an active area of research.

## Therapies

### Ventilation

Ventilation of neurologically injured patients entails a balance between preventing intracranial pressure elevation secondary to hypercarbia while minimizing exposure to an injurious factor. The association between high-tidal-volume ventilation and ARDS in patients with intracerebral hemorrhage highlights the importance of incorporating tidal volume minimization in ventilation setting strategies [24]. Animal studies comparing low- versus high-tidal-volume ventilation in porcine ARDS models find improved oxygenation and lower lactate levels in brain tissue when using low-tidal-volume ventilation, which is supported by clinical outcomes in human patients [52, 82]. Patients ventilated at lower tidal volumes (≤ 8 mL/kg) following cardiac arrest had a significantly higher chance of being classified as cerebral performance category 1 at follow-up [82]. Avoidance of benzodiazepines and selection of dexmedetomidine as a sedative agent may reduce the risk of delirium in patients with ARDS regardless of neurological injury status [46].

Given the systemic inflammatory effects of ARDS, in addition to murine studies implicating TNF-α in mediating neutrophil recruitment in the early phases of high-tidal-volume-associated stretch lung injury, immunomodulating therapies such as monoclonal antibodies against TNF-α may represent a future area of pharmacotherapy [83]. As the receptor for advanced glycation end products (RAGE) has been found to mediate amyloid-β influx and microglial activation at the blood-brain barrier, this may also represent a future pharmacological target in humans, with data from mouse models of Alzheimer’s disease showing effective control of amyloid-β accumulation and amyloid-β-mediated cellular stress using RAGE inhibitors [84].

### Fluid management

Fluid management reflects another vital juncture of consideration, particularly among neurologically injured patients with concurrent sepsis. The Fluid and Catheter Treatment Trial found that despite similar 60-day mortality, patients with ARDS treated with conservative rather than liberal fluid management (central venous pressure < 4 versus 10–14, respectively) experience shorter intensive care unit stays and fewer days requiring a ventilator [85]. However, follow-up of this trial found an association between conservative fluid management and worse cognitive function [1]. The choice of whether to target treatment toward cerebral perfusion pressure or intracranial pressure goals in the management of brain injury invites further consideration of fluid management strategy in this patient population. A study comparing treatment strategies in patients with severe traumatic brain injury found a significantly elevated risk of developing ARDS among patients receiving cerebral perfusion pressure-targeted treatment compared to those receiving intracranial pressure-targeted treatment, with an odds ratio of 5.1 [19]. Patients with brain injuries appear uniquely susceptible to deleterious effects of vasopressors: when vasopressors were used in order to meet cerebral perfusion pressure goals, the risk of developing ARDS further rose, with odds ratios of 5.7 with epinephrine (95% CI 1.0375–34.3323) and 10.8 with dopamine (95% CI 1.4–488.3) [19]. The widespread use of high PEEP in ARDS raises questions of whether interactions between PEEP and intracranial pressure or cerebral perfusion pressure require changes in practice when treating neurologically injured patients who do go on to develop ARDS. Studies investigating the relationship between PEEP and cerebral microcirculation identify hemodynamic stability as an important factor mitigating against adverse effects of PEEP [79, 80]. Specifically, despite adverse microcirculatory effects of PEEP, Muench et al. found that it was only in the scenario of existing hemodynamic instability that PEEP adversely decreased cerebral perfusion pressure [80], and Georgiadis et al. similarly found that PEEP did not affect intracranial pressure as long as mean arterial pressure remained stable [79]. Therefore, while fluid minimization may be beneficial in a general population of critically ill patients [85], aggressive application of a conservative fluid management approach in neurologically injured patients—especially if vasopressors become required to maintain hemodynamic stability—may contribute to not only the risk of ARDS, but also that of cerebral hypoperfusion after initiating treatment with high-PEEP ventilation. Future studies are needed to determine an optimal, perhaps individualized PEEP threshold, that maximizes pulmonary and neurological function.

### Other interventions

The interdisciplinary, collaborative environment of the intensive care unit allows for coordinated patient care optimization approaches to mitigate contributors to patient discomfort and long-term adverse effects. These interventions not only are relevant for neurologically injured cohorts of patients with ARDS, but may also improve outcomes in the general population of intensive care unit patients. A recent example of such an approach is the ABCDEF bundle, comprising the following elements: assess, prevent, and manage pain; both spontaneous awakening trials and spontaneous breathing trials; choice of analgesia and sedation; delirium: assess, prevent, and manage; early mobility and exercise; and family engagement and empowerment [86]. Implementation of the ABCDEF bundle for 15,000 patients as part of the ICU Liberation Collaborative found that patients treated with this bundle experienced significant differences in outcomes compared to those who did not, including higher likelihoods of discharge home and survival within the first 7 days of hospitalization, and lower likelihoods of coma, delirium, physical restraint use, ventilator-dependence, and readmission [86]. Improvements in each of these outcomes showed a significant dose-response corresponding with compliance with the bundle, in which 10% increases in bundle compliance produced 15% improvement in survival and days without coma and delirium [86]. Minimizing corticosteroid use and length of stay may also reduce the risk of adverse long-term cognitive and physical outcomes among ARDS survivors [6].

## Conclusion

Reducing the practical burden of cognitive recovery following critical illness depends crucially on understanding the links between brain injury and lung injury. New deficits in learning and memory, and new development of psychiatric illness, inherently limit the maximum extent of recovery by restricting the extent to which patients can meaningfully participate in rehabilitation. The influence of pre-existing cognitive impairment on susceptibility and recovery highlights the importance of accurate detection and definition of cognitive impairment. Evidence for distinct patterns of inflammatory damage, the predilection of cytokines for the hippocampus, and activation of systemic inflammatory pathways in high-tidal-volume mechanical ventilation collectively support minimizing tidal volume as much as possible to avoid ARDS, in turn helping prevent further endothelial and microglial activation of the inflammatory cascade. The ability to control and monitor parameters such as PEEP and fluid balance in the intensive care unit setting provides an opportunity to optimize treatment on an individual basis to minimize the risk of long-term cognitive impairment.

As new data continue to reveal increasing layers of complexity with which these parameters influence the brain-lung axis and one another, further studies will hope to elucidate management strategies that entail not just minimization or maximization of individual variables, but a balanced and nuanced approach to achieve both physical and cognitive recovery from ARDS. Mindful implementation of multidisciplinary approaches to providing more humanistic patient care may improve long-term cognitive outcomes.

## Data Availability

Not applicable

## Abbreviations

ARDS: Acute respiratory distress syndrome
CAM-ICU: Confusion Assessment Method for Intensive Care Unit Patients
PEEP: Positive end-expiratory pressure
RAGE: Receptor for advanced glycation end products
ABCDEF: Assess, prevent, and manage pain; both spontaneous awakening trials and spontaneous breathing trials; choice of analgesia and sedation; delirium: assess, prevent, and manage; early mobility and exercise; and family engagement and empowerment

## References

[1] Mikkelsen ME, Christie JD, Lanken PN, Biester RC, Taylor Thompson B, Bellamy SL (2012). The adult respiratory distress syndrome cognitive outcomes study: long-term neuropsychological function in survivors of acute lung injury. Am Thoracic Soc.

[2] Bellani G, Laffey JG, Pham T, Fan E, Brochard L, Esteban A (2016). Epidemiology, patterns of care, and mortality for patients with acute respiratory distress syndrome in intensive care units in 50 countries. JAMA..

[3] Herridge MS, Moss M, Hough CL, Hopkins RO, Rice TW, Bienvenu OJ (2016). Recovery and outcomes after the acute respiratory distress syndrome (ARDS) in patients and their family caregivers. Intensive Care Med.

[4] Wilcox ME, Brummel NE, Archer K, Ely EW, Jackson JC, Hopkins RO (2013). Cognitive dysfunction in ICU patients: risk factors, predictors, and rehabilitation interventions. Crit Care Med.

[5] Davidson TA, Caldwell ES, Curtis JR, Hudson LD, Steinberg KP (1999). Reduced quality of life in survivors of acute respiratory distress syndrome compared with critically ill control patients. JAMA..

[6] Needham DM, Wozniak AW, Hough CL, Morris PE, Dinglas VD, Jackson JC (2014). Risk factors for physical impairment after acute lung injury in a national, multicenter study. Am J Respir Crit Care Med.

[7] Herridge MS, Tansey CM, Matté A, Tomlinson G, Diaz-Granados N, Cooper A (2011). Functional disability 5 years after acute respiratory distress syndrome. N Engl J Med.

[8] Mikkelsen ME, Shull WH, Biester RC, Taichman DB, Lynch S, Demissie E (2009). Cognitive, mood and quality of life impairments in a select population of ARDS survivors. Respirology..

[9] Kapfhammer HP, Rothenhäusler HB, Krauseneck T, Stoll C, Schelling G (2004). Posttraumatic stress disorder and health-related quality of life in long-term survivors of acute respiratory distress syndrome. Am J Psychiatr.

[10] Hopkins RO, Weaver LK, Collingridge D, Parkinson RB, Chan KJ, Orme JF (2005). Two-year cognitive, emotional, and quality-of-life outcomes in acute respiratory distress syndrome. Am J Respir Crit Care Med.

[11] Lee HB, DeLoatch CJ, Cho S, Rosenberg P, Mears SC, Sieber FE (2008). Detection and management of pre-existing cognitive impairment and associated behavioral symptoms in the intensive care unit. Crit Care Clin.

[12] Ely EW, Inouye SK, Bernard GR, Gordon SM, Francis J, May L (2001). Delirium in mechanically ventilated patients. JAMA..

[13] Pisani MA, Inouye SK, McNicoll L, Redlich CA (2003). Screening for preexisting cognitive impairment in older intensive care unit patients: use of proxy assessment. J Am Geriatr Soc.

[14] Pisani MA, Redlich C, McNicoll L, Ely EW, Inouye SK (2003). Underrecognition of preexisting cognitive impairment by physicians in older ICU patients. CHEST J.

[15] Pfoh ER, Chan KS, Dinglas VD, Girard TD, Jackson JC, Morris PE (2015). Cognitive screening among acute respiratory failure survivors: a cross-sectional evaluation of the Mini-Mental State Examination. Crit Care.

[16] Oddo M, Citerio G (2016). ARDS in the brain-injured patient: what’s different?. Intensive Care Med.

[17] Holland MC, Mackersie RC, Morabito D, Campbell AR, Kivett VA, Patel R (2003). The development of acute lung injury is associated with worse neurologic outcome in patients with severe traumatic brain injury. J Trauma.

[18] Stevens RD, Puybasset L (2011). The brain-lung-brain axis. Intensive Care Med.

[19] Contant CF, Valadka AB, Gopinath SP, Hannay HJ, Robertson CS (2001). Adult respiratory distress syndrome: a complication of induced hypertension after severe head injury. J Neurosurg.

[20] Mascia L, Andrews PJ (1998). Acute lung injury in head trauma patients. Intensive Care Med.

[21] Mascia L, Zavala E, Bosma K, Pasero D, Decaroli D, Andrews P (2007). High tidal volume is associated with the development of acute lung injury after severe brain injury: an international observational study. Crit Care Med.

[22] Piek J, Chesnut RM, Marshall LF, van Berkum-Clark M, Klauber MR, Blunt BA (1992). Extracranial complications of severe head injury. J Neurosurg.

[23] Bronchard R, Albaladejo P, Brezac G, Geffroy A, Seince P-F, Morris W (2004). Early onset pneumonia: risk factors and consequences in head trauma patients. Anesthesiology..

[24] Elmer J, Hou P, Wilcox SR, Chang Y, Schreiber H, Okechukwu I (2013). Acute respiratory distress syndrome after spontaneous intracerebral hemorrhage*. Crit Care Med.

[25] Gross AL, Jones RN, Habtemariam DA, Fong TG, Tommet D, Quach L (2012). Delirium and long-term cognitive trajectory among persons with dementia. Arch Intern Med.

[26] Pandharipande PP, Girard TD, Jackson JC, Morandi A, Thompson JL, Pun BT (2013). Long-term cognitive impairment after critical illness. N Engl J Med.

[27] Danysz W, Parsons CG (2012). Alzheimer’s disease, β-amyloid, glutamate, NMDA receptors and memantine - searching for the connections. Br J Pharmacol.

[28] Takahashi RH, Nagao T, Gouras GK (2017). Plaque formation and the intraneuronal accumulation of β-amyloid in Alzheimer’s disease. Pathol Int.

[29] Ehlenbach WJ, Hough CL, Crane PK, Haneuse SJPA, Carson SS, Curtis JR (2010). Association between acute care and critical illness hospitalization and cognitive function in older adults. JAMA..

[30] Girard TD, Thompson JL, Pandharipande PP, Brummel NE, Jackson JC, Patel MB (2018). Clinical phenotypes of delirium during critical illness and severity of subsequent long-term cognitive impairment: a prospective cohort study. Lancet Respir Med.

[31] McNicoll L, Pisani MA, Zhang Y, Ely EW, Siegel MD, Inouye SK (2003). Delirium in the intensive care unit: occurrence and clinical course in older patients. J Am Geriatr Soc.

[32] Pisani MA, Murphy TE, Van Ness PH, Araujo KLB, Inouye SK (2007). Characteristics associated with delirium in older patients in a medical intensive care unit. Arch Intern Med.

[33] Ely EW, Shintani A, Truman B, Speroff T, Gordon SM, Harrell FE (2004). Delirium as a predictor of mortality in mechanically ventilated patients in the intensive care unit. JAMA..

[34] Witlox J, Eurelings LSM, de Jonghe JFM, Kalisvaart KJ, Eikelenboom P, van Gool WA (2010). Delirium in elderly patients and the risk of postdischarge mortality, institutionalization, and dementia: a meta-analysis. JAMA..

[35] Fong TG, Jones RN, Shi P, Marcantonio ER, Yap L, Rudolph JL (2009). Delirium accelerates cognitive decline in Alzheimer disease. Neurology..

[36] Davis DHJ, Muniz-Terrera G, Keage HAD, Stephan BCM, Fleming J, Ince PG (2017). Association of delirium with cognitive decline in late life: a neuropathologic study of 3 population-based cohort studies. JAMA Psychiatry.

[37] Davis DHJ, Skelly DT, Murray C, Hennessy E, Bowen J, Norton S (2015). Worsening cognitive impairment and neurodegenerative pathology progressively increase risk for delirium. Am J Geriatr Psychiatr.

[38] MacLullich AMJ, Ferguson KJ, Miller T, de Rooij SEJA, Cunningham C (2008). Unravelling the pathophysiology of delirium: a focus on the role of aberrant stress responses. J Psychosom Res.

[39] van den Boogaard M, Kox M, Quinn KL, van Achterberg T, van der Hoeven JG, Schoonhoven L (2011). Biomarkers associated with delirium in critically ill patients and their relation with long-term subjective cognitive dysfunction; indications for different pathways governing delirium in inflamed and noninflamed patients. Crit Care.

[40] Hopkins RO, Weaver LK, Pope D, Orme JF, Bigler ED, Larson-LOHR V (1999). Neuropsychological sequelae and impaired health status in survivors of severe acute respiratory distress syndrome. Am J Respir Crit Care Med.

[41] Dantzer R (2001). Cytokine-induced sickness behavior: mechanisms and implications. Ann N Y Acad Sci.

[42] Harrison NA, Brydon L, Walker C, Gray MA, Steptoe A, Dolan RJ (2009). Neural origins of human sickness in interoceptive responses to inflammation. Biol Psychiatry.

[43] Cunningham C, MacLullich AMJ (2013). At the extreme end of the psychoneuroimmunological spectrum: delirium as a maladaptive sickness behaviour response. Brain Behav Immun.

[44] Jackson JC, Hart RP, Gordon SM, Shintani A, Truman B, May L (2003). Six-month neuropsychological outcome of medical intensive care unit patients. Crit Care Med.

[45] Lahiri S, Regis GC, Koronyo Y, Fuchs DT, Sheyn J, Kim EH (2019). Acute neuropathological consequences of short-term mechanical ventilation in wild-type and Alzheimer’s disease mice. Crit Care.

[46] Shah FA, Girard TD, Yende S (2017). Limiting sedation for patients with acute respiratory distress syndrome - time to wake up. Curr Opin Crit Care.

[47] Evered L, Silbert B, Scott DA, Zetterberg H, Blennow K (2018). Association of changes in plasma neurofilament light and tau levels with anesthesia and surgery. JAMA Neurol.

[48] Matute-Bello G, Frevert CW, Martin TR (2008). Animal models of acute lung injury. Am J Phys Lung Cell Mol Phys.

[49] Wolthuis EK, Vlaar APJ, Choi G, Roelofs JJTH, Juffermans NP, Schultz MJ (2009). Mechanical ventilation using non-injurious ventilation settings causes lung injury in the absence of pre-existing lung injury in healthy mice. Crit Care.

[50] Fries M, Bickenbach J, Henzler D, Beckers S, Dembinski R, Sellhaus B (2005). S-100 protein and neurohistopathologic changes in a porcine model of acute lung injury. Anesthesiology..

[51] Bickenbach J, Biener I, Czaplik M, Nolte K, Dembinski R, Marx G (2011). Neurological outcome after experimental lung injury. Respir Physiol Neurobiol.

[52] Bickenbach J, Zoremba N, Fries M, Dembinski R, Doering R, Ogawa E (2009). Low tidal volume ventilation in a porcine model of acute lung injury improves cerebral tissue oxygenation. Anesth Analg.

[53] Dai S-S, Wang H, Yang N, An J-H, Li W, Ning Y-L (2013). Plasma glutamate–modulated interaction of A2AR and mGluR5 on BMDCs aggravates traumatic brain injury–induced acute lung injury. J Exp Med.

[54] Winklewski PJ, Radkowski M, Demkow U (2014). Cross-talk between the inflammatory response, sympathetic activation and pulmonary infection in the ischemic stroke. J Neuroinflammation.

[55] Sonneville R, Verdonk F, Rauturier C, Klein IF, Wolff M, Annane D (2013). Understanding brain dysfunction in sepsis. Ann Intensive Care.

[56] Taccone FS, Castanares-Zapatero D, Peres-Bota D, Vincent J-L, Berre J, Melot C (2009). Cerebral autoregulation is influenced by carbon dioxide levels in patients with septic shock. Neurocrit Care.

[57] Wang J, Gu BJ, Masters CL, Wang Y-J (2017). A systemic view of Alzheimer disease - insights from amyloid-β metabolism beyond the brain. Nat Rev Neurol.

[58] Montagne A, Zhao Z, Zlokovic BV (2017). Alzheimer’s disease: a matter of blood-brain barrier dysfunction?. J Exp Med.

[59] Iliff JJ, Wang M, Liao Y, Plogg BA, Peng W, Gundersen GA (2012). A paravascular pathway facilitates CSF flow through the brain parenchyma and the clearance of interstitial solutes, including amyloid beta. Sci Transl Med.

[60] Erickson MA, Banks WA (2013). Blood–brain barrier dysfunction as a cause and consequence of Alzheimer’s disease. J Cerebral Blood Flow Metab.

[61] Song J, Choi S-M, Whitcomb DJ, Kim BC (2017). Adiponectin controls the apoptosis and the expression of tight junction proteins in brain endothelial cells through AdipoR1 under beta amyloid toxicity. Cell Death Dis.

[62] Miguel-Hidalgo JJ, Cacabelos R (1998). Beta-amyloid(1-40)-induced neurodegeneration in the rat hippocampal neurons of the CA1 subfield. Acta Neuropathol.

[63] Freir DB, Holscher C, Herron CE (2001). Blockade of long-term potentiation by beta-amyloid peptides in the CA1 region of the rat hippocampus in vivo. J Neurophysiol.

[64] Hopkins RO, Gale SD, Weaver LK (2006). Brain atrophy and cognitive impairment in survivors of acute respiratory distress syndrome. Brain Inj.

[65] Janz DR, Abel TW, Jackson JC, Gunther ML, Heckers S, Ely EW (2010). Brain autopsy findings in intensive care unit patients previously suffering from delirium: a pilot study. J Crit Care.

[66] Montagne A, Barnes SR, Sweeney MD, Halliday MR, Sagare AP, Zhao Z (2015). Blood-brain barrier breakdown in the aging human hippocampus. Neuron..

[67] Barrientos RM, Higgins EA, Biedenkapp JC, Sprunger DB, Wright-Hardesty KJ, Watkins LR (2006). Peripheral infection and aging interact to impair hippocampal memory consolidation. Neurobiol Aging.

[68] Chen J, Buchanan JB, Sparkman NL, Godbout JP, Freund GG, Johnson RW (2008). Neuroinflammation and disruption in working memory in aged mice after acute stimulation of the peripheral innate immune system. Brain Behav Immun.

[69] Semmler A, Okulla T, Sastre M, Dumitrescu-Ozimek L, Heneka MT (2005). Systemic inflammation induces apoptosis with variable vulnerability of different brain regions. J Chem Neuroanat.

[70] Harry GJ, Lefebvre d’Hellencourt C (2003). Dentate gyrus: alterations that occur with hippocampal injury. NeuroToxicology..

[71] Imamura Y, Wang H, Matsumoto N, Muroya T, Shimazaki J, Ogura H (2011). Interleukin-1β causes long-term potentiation deficiency in a mouse model of septic encephalopathy. Neuroscience..

[72] Shankar GM, Li S, Mehta TH, Garcia-Munoz A, Shepardson NE, Smith I (2008). Amyloid-beta protein dimers isolated directly from Alzheimer’s brains impair synaptic plasticity and memory. Nat Med.

[73] Zlokovic BV (2011). Neurovascular pathways to neurodegeneration in Alzheimer’s disease and other disorders. Nat Rev Neurosci.

[74] Shohami E, Novikov M, Bass R, Yamin A, Gallily R (2016). Closed head injury triggers early production of TNFα and IL-6 by brain tissue. J Cerebral Blood Flow Metab.

[75] López-Aguilar J, Villagrá A, Bernabé F, Murias G, Piacentini E, Real J (2005). Massive brain injury enhances lung damage in an isolated lung model of ventilator-induced lung injury. Crit Care Med.

[76] Imai Y, Parodo J, Kajikawa O, de Perrot M, Fischer S, Edwards V (2003). Injurious mechanical ventilation and end-organ epithelial cell apoptosis and organ dysfunction in an experimental model of acute respiratory distress syndrome. JAMA..

[77] Ko GJ, Rabb H, Hassoun HT (2009). Kidney-lung crosstalk in the critically ill patient. Blood Purif.

[78] van den Akker JP, Egal M, Groeneveld JA. Invasive mechanical ventilation as a risk factor for acute kidney injury in the critically ill: a systematic review and meta-analysis. Crit Care. 2013;17(3):R98. 10.1186/cc12743 PMC3706893 23710662

[79] Georgiadis D, Schwarz S, Baumgartner RW, Veltkamp R, Schwab S (2001). Influence of positive end-expiratory pressure on intracranial pressure and cerebral perfusion pressure in patients with acute stroke. Stroke..

[80] Muench E, Bauhuf C, Roth H, Horn P, Phillips M, Marquetant N (2005). Effects of positive end-expiratory pressure on regional cerebral blood flow, intracranial pressure, and brain tissue oxygenation*. Crit Care Med.

[81] Mascia L, Grasso S, Fiore T, Bruno F, Berardino M, Ducati A (2005). Cerebro-pulmonary interactions during the application of low levels of positive end-expiratory pressure. Intensive Care Med.

[82] Beitler JR, Ghafouri TB, Jinadasa SP, Mueller A, Hsu L, Anderson RJ (2017). Favorable neurocognitive outcome with low tidal volume ventilation after cardiac arrest. Am J Respir Crit Care Med.

[83] Wilson MR, Choudhury S, Takata M (2005). Pulmonary inflammation induced by high-stretch ventilation is mediated by tumor necrosis factor signaling in mice. Am J Phys Lung Cell Mol Phys.

[84] Deane R, Singh I, Sagare AP, Bell RD, Ross NT, LaRue B (2012). A multimodal RAGE-specific inhibitor reduces amyloid beta-mediated brain disorder in a mouse model of Alzheimer disease. J Clin Invest.

[85] National Heart L, and Blood Institute Acute Respiratory Distress Syndrome (ARDS) Clinical Trials Network, Wiedemann HP, Wheeler AP, Bernard GR, Thompson BT, Hayden D, et al. Comparison of two fluid-management strategies in acute lung injury. N Engl J Med 2006;354(24):2564–2575. 10.1056/NEJMoa062200 16714767

[86] Pun BT, Balas MC, Barnes-Daly MA, Thompson JL, Aldrich JM, Barr J (2019). Caring for critically ill patients with the ABCDEF bundle: results of the ICU liberation collaborative in over 15,000 adults. Crit Care Med.

